# COVID-19-associated apnea and circumoral cyanosis in a 3-week-old

**DOI:** 10.1186/s12887-020-02282-8

**Published:** 2020-08-12

**Authors:** Joseph S. Needleman, Amy E. Hanson

**Affiliations:** 1 Internal Medicine–Pediatrics Residency Program, Indiana University School of Medicine, 705 Riley Hospital Drive, Rm 5837, Indianapolis, IN 46202 USA; 2 Pediatric Residency Program, Indiana University School of Medicine, Indianapolis, IN USA

**Keywords:** COVID-19, SARS-CoV-2, Apnea, Cyanosis, BRUE, Neonate, Infant, Children, Pediatrics

## Abstract

**Background:**

Data regarding coronavirus disease 2019 (COVID-19) cases and outcomes in infants are sparse compared to older pediatric and adult populations.

**Case presentation:**

We present a three-week-old full-term male with a history of mild hypoxic ischemic encephalopathy (HIE) who was admitted as an inpatient twice for episodes of apnea and perioral cyanosis. The patient tested positive for COVID-19 and negative for other common respiratory viruses at both admissions.

**Conclusions:**

To our knowledge, this is the first report of apnea and perioral cyanosis associated with COVID-19 in an infant. This case highlights a previously undocumented COVID-19 presentation and suggests that even mildly symptomatic infants warrant viral diagnostic testing in an effort to prevent further spread of the disease.

## Background

The world is in the midst of a global pandemic due to COVID-19, an infectious disease caused by severe acute respiratory syndrome coronavirus 2 (SARS-CoV-2). Due to its novelty there is still limited knowledge about its natural history, and data regarding its effect on pediatric patients is particularly sparse compared to adult patients [1]. From what is currently understood, in the pediatric population, particularly in younger children, morbidity and mortality rates are notably lower than the adult population [2]. The most common clinical features of COVID-19 in children are fever, cough, and fatigue, which are symptoms also associated with numerous common respiratory tract infections. Younger patients usually require only supportive care and generally recover fully within two weeks of symptom onset [3–5], though there have been multiple anecdotal reports of COVID-19 related deaths in infants. We present a case of apnea and perioral cyanosis, initially worked up as a brief resolved unexplained event (BRUE), associated with COVID-19.

## Case presentation

A 25-day-old full-term male infant with a history of mild HIE was admitted initially for work-up of BRUE. His guardians described a single episode three days prior to presentation during which the patient stopped breathing and developed perioral cyanosis for approximately 3–4 s while sleeping, which resolved with mild stimulation and waking. Associated symptoms included nasal congestion and rhinorrhea. Multiple family members had been experiencing fevers, cough, and congestion starting the week prior. The patient’s only additional medical history was respiratory distress at birth requiring 24 h of positive pressure ventilation, and episodes of desaturation during his first six days of life which were attributed to possible mild laryngomalacia.

Vital signs were within normal limits. Physical exam was without abnormal findings. A nasopharyngeal and oropharyngeal polymerase chain reaction (PCR) test for SARS-CoV-2 and a respiratory viral panel (RVP) PCR were collected. Continuous pulse oximetry overnight was normal, and he was discharged home the subsequent morning with instructions to presumptively quarantine while RVP and COVID-19 testing were pending. Shortly after discharge, his COVID-19 PCR resulted positive and RVP resulted negative.

Three weeks after his initial presentation, at 45 days of life, the patient was brought back to the hospital after his guardians that day noted new recurrent episodes of apnea and perioral cyanosis, intermittent stridor, and abnormal head and eye movements. Physical exam was unchanged from his prior admission except for intermittent inspiratory stridor noted while the patient was asleep. A basic metabolic panel was within normal limits and repeat COVID-19 and RVP PCR were positive and negative, respectively. He was evaluated by a speech therapist who did not feel he was at risk for aspiration. Neurology was consulted, and after reviewing his prior normal electroencephalograms and non-concerning brain MRI felt that, in the context of his mild HIE diagnosis and age-appropriate movements, his history was not consistent with seizures. No further apneic or cyanotic episodes were noted, and vital signs and pulse oximetry remained stable, so he was discharged within 24 h of admission with scheduled outpatient neurology and pulmonology follow-ups.

## Discussion and conclusion

Data about COVID-19 in the infant population is scarce. Less than 1% of patients in a review of over 72,000 cases from China were younger than 10 years of age, and less than 20% of a pediatric subset were younger than 1 year of age [6, 7]. There is contradictory evidence regarding vertical transmission of COVID-19 between infected mothers and newborns, though the current consensus is that vertical transmission is unlikely to occur [8, 9]. Symptoms in this age group vary as with older age groups. A case series of nine hospitalized patients under the age of one noted that most hospitalized patients were symptomatic with fever and upper respiratory symptoms [10]. Another reported case described a 55-day-old infant who developed more severe symptoms, including pneumonia, and demonstrated evidence of liver and cardiac injury [11].

In younger populations, viral load may persist for weeks regardless of symptoms, as case reports have noted persistently positive PCR tests even in asymptomatic patients, including in a well 6-month-old who tested positive for 16 days [12]. As our patient’s immediate family members were sick one week prior to the onset of his symptoms, his family was the likely source from which he contracted the virus. He tested positive on admission for both hospitalizations via PCR testing, with 21 days between the two tests.

Our patient’s presentation, initially for an isolated episode of perioral cyanosis and apnea, was at first attributed to a BRUE. A BRUE is defined as an event lasting less than one minute without an identifiable explanation and with full resolution of symptoms, in an infant less than one year of age, and symptoms can include cyanosis [13]. Past research has found a strong correlation between apparent life-threatening events, a less-specific diagnosis that has been replaced by the term “BRUE,” and a positive respiratory viral infection test [13]. However, given his recurrence of symptoms and new onset of stridor in the setting of a persistently positive COVID-19 PCR, his symptoms are more likely to be directly due to his infection by SARS-CoV-2. Furthermore, the recurrence of symptoms after a prolonged asymptomatic period indicates this patient’s infection appears to have followed an atypical course.

Despite increasing evidence demonstrating children have more mild presentations and better outcomes in COVID-19 infections, there is limited documentation of individual cases, especially infants, in the medical literature. Since children appear to most commonly present asymptomatically or mildly symptomatic, they have the potential to serve as undetected vectors of the disease as cities and countries start to loosen restrictions on commerce and socialization. Given the lack of universal testing infrastructure worldwide, the implication therefore may be to focus on opportunities to test these patients whenever possible. With disease modeling estimating continued COVID-19 infection in the years ahead, our case highlights the need for clinicians to consider more subtle presentations in infants such as brief episodes of apnea, cyanosis, and stridor as potential catalysts to prompt testing for SARS-CoV-2 infection, caregiver testing, and/or recommending quarantine protocol.

## Data Availability

Not applicable.

## Abbreviations

COVID-19: Coronavirus Disease 2019
HIE: Hypoxic ischemic encephalopathy
BRUE: Brief resolved unexplained event (BRUE)
SARS-CoV-2: Severe acute respiratory syndrome coronavirus 2
PCR: Polymerase chain reaction
RVP: Respiratory viral panel
